# Effects of Germinated Brown Rice and Its Bioactive Compounds on the Expression of the Peroxisome Proliferator-Activated Receptor Gamma Gene

**DOI:** 10.3390/nu5020468

**Published:** 2013-02-06

**Authors:** Mustapha Umar Imam, Maznah Ismail, Hairuszah Ithnin, Zaki Tubesha, Abdul Rahman Omar

**Affiliations:** 1 Laboratory of Molecular Biomedicine, Institute of Bioscience, Universiti Putra Malaysia, Serdang, Selangor 43400, Malaysia; E-Mails: mustyimam@gmail.com (M.U.I.); zak33us@yahoo.com (Z.T.); aro@ibs.upm.edu.my (A.R.O.); 2 Department of Nutrition and Dietetics, Faculty of Medicine and Health Sciences, Universiti Putra Malaysia, Serdang, Selangor 43400, Malaysia; 3 Department of Pathology, Faculty of Medicine and Health Sciences, Universiti Putra Malaysia, Serdang, Selangor 43400, Malaysia; E-Mail: hairusza@medic.upm.edu.my; 4 Department of Veterinary Pathology & Microbiology, Faculty of Veterinary Medicine, Universiti Putra Malaysia, Serdang, Selangor 43400, Malaysia

**Keywords:** peroxisome proliferator activated receptor gamma, germinated brown rice, oryzanol, gamma amino butyric acid, acylated steryl glycoside

## Abstract

Dysregulated metabolism is implicated in obesity and other disease conditions like type 2 diabetes mellitus and cardiovascular diseases, which are linked to abnormalities of peroxisome proliferator-activated receptor gamma (*PPARγ*). *PPARγ* has been the focus of much research aimed at managing these diseases. Also, germinated brown rice (GBR) is known to possess antidiabetic, antiobesity and hypocholesterolemic effects. We hypothesized that GBR bioactive compounds may mediate some of the improvements in metabolic indices through *PPARγ* modulation. Cultured HEP-G2 cells were treated with 50 ppm and 100 ppm of extracts from GBR (GABA, ASG and oryzanol) after determination of cell viabilities using MTT assays. Results showed that all extracts upregulated the expression of the *PPARγ*. However, combination of all three extracts showed downregulation of the gene, suggesting that, in combination, the effects of these bioactives differ from their individual effects likely mediated through competitive inhibition of the gene. Upregulation of the gene may have therapeutic potential in diabetes mellitus and cardiovascular diseases, while its downregulation likely contributes to GBR’s antiobesity effects. These potentials are worth studying further.

## 1. Introduction

Germinated brown rice (GBR) is a product of biotransformation of brown rice (BR), and it has been shown to contain high amounts of bioactives with health promoting benefits [1,2]. GBR has antidiabetic, hypocholesterolemic, antiobesity and other properties attributed to the presence of its bioactive compounds. Food synergy, due to contribution of the different bioactive compounds in GBR, may likely play a role in its overall functional effects [3]. Since these bioactive compounds likely confer GBR with its functionality, and some of them have similar effects on metabolic indices, it is likely that they elicit complex biological interactions.

Better understanding of molecular targets involved in GBR’s effects may create opportunities to explore the use of GBR or its bioactives as a functional food component or potential nutraceutical, for management and/or prevention of diseases that result from breakdown of the pathways regulated by the molecular targets. Potential molecular targets implicated in many disease processes are peroxisome proliferator-activated receptors (PPARs), which are nuclear receptor proteins involved in a wide variety of regulatory functions [4], making them important targets in the management of metabolic conditions [5]. Dysregulation of PPAR-gamma (*PPARγ*) is linked to the development of obesity, type 2 diabetes, atherosclerosis and other disease conditions [6]. To improve its activity, mostly agonists have been used [7]. Its ligands may cause antiatherogenesis through reducing inflammatory changes thereby reducing risk of atherosclerosis and cardiovascular events [8]. In addition, dietary components may upregulate the expression of the *PPARγ* as their mechanism of action towards disease prevention [9]. Interestingly, GBR’s antiobesity effect was found to be mediated partly through downregulation of the *PPARγ* [10]. These contrasting effects point to the interesting and varied functional effects of *PPARγ*; both upregulation and downregulation of the gene have been reported to be beneficial in different disease conditions [8,9,10]. Increasing concern for side effects such as heptotoxicity and cardiotoxicity associated with *PPARγ* agonists has necessitated the need for better alternatives [7,11,12]. Interestingly, many dietary components like tocopherol, linoleic acid, curcumin and resveratrol upregulate *PPARγ* expression with no reported side effects in chronic diseases like obesity, cardiovascular diseases, colon cancer, and diabetes [11]. 

Thus, we studied the effects of gamma-amino butyric acid (GABA), oryzanol, and acylated steryl glycoside (ASG) extracts from GBR as potential regulators of *PPARγ*, in order to determine their nutraceutical potential in the management of diseases resulting from *PPARγ* dysregulation. The analysis involved a multiplex panel with *PPARγ*, *3-hydroxy-3-methyl-glutaryl-CoA reductase* (*HMGCR*) and *insulin receptor* (*IR*) genes, in order to determine the effects of these extracts, if any, on key aspects of cholesterol metabolism (*HMGCR*) and insulin signaling (*IR*), in addition to *PPARγ* regulation. 

## 2. Experimental Section

### 2.1. Materials

GABA, oryzanol and phenolics standards, streptozotocin (STZ), Tris-EDTA (TE) buffer solution, dexamethasone, *N*,*O*-bis(trimethylsilyl)trifluoroacetamide with 1% Trimethylchlorosilane (BSTFA + 1% TMCS), RPMI 1640 medium, fetal bovine serum, and antibiotic were purchased from Sigma-Aldrich (St. Louis, Missouri, USA). The ASG standard was purchased from Matreya (Pleasant Gap, PA, USA), and MgCl_2 _and DNA Taq polymerase were purchased from Thermo Fisher Scientific (Pittsburgh, PA, USA). Insulin was purchased from Invitrogen (Carlsbad, CA, USA) and other cell cultureware was purchased from BD Bioscience (NJ, USA). The GenomeLab™ GeXP Start Kit was from Beckman Coulter Inc. (Miami, FL, USA), while the Total RNA isolation kit was supplied by RBC Bioscience Corp. (Taipei, Taiwan). Hydrogen peroxide (H_2_O_2_) was from Bendosen Laboratory Chemicals (Selangor, Malaysia) and sodium hypochlorite from Dexchem Industries Sdn. Bhd, (Penang, Malaysia). All solvents of analytical grade were purchased from Merck (Darmstadt, Germany).

### 2.2. Rice Samples

BR of Malaysian rice variety (MR220) was supplied by PadiBeras Nasional (BERNAS) (Selangor, Malaysia) and germinated as reported in an earlier publication [13].

### 2.3. Preparation of Extracts

Oryzanol, GABA and ASG extracts from GBR were used in this study. Oryzanol was prepared and analyzed as reported by Azlan *et al.* (2008) [14], and GABA was extracted and analyzed on HPLC-DAD (Agilent, Santa Clara, CA) as reported by Rozan *et al.* (2000) [15]. ASG was extracted as reported by Usuki *et al.* (2008) [16], and analyzed on GC-MS/MS QqQ (Thermo Fischer Scientific, Logan, UT) following the method of Philip *et al.* (2005) [17]. 

### 2.4. Cell Culture

HEP-G2 cells were acquired from the American Type Culture Collection (Manassas, VA) and cultured in RPMI 1640 medium supplemented with 10% fetal bovine serum (FBS) and 1% antibiotics (100 U/mL penicillin) in an incubator at 37 °C with 5% CO_2_. 

### 2.5. Cell Viability Assay

**Figure 1:**
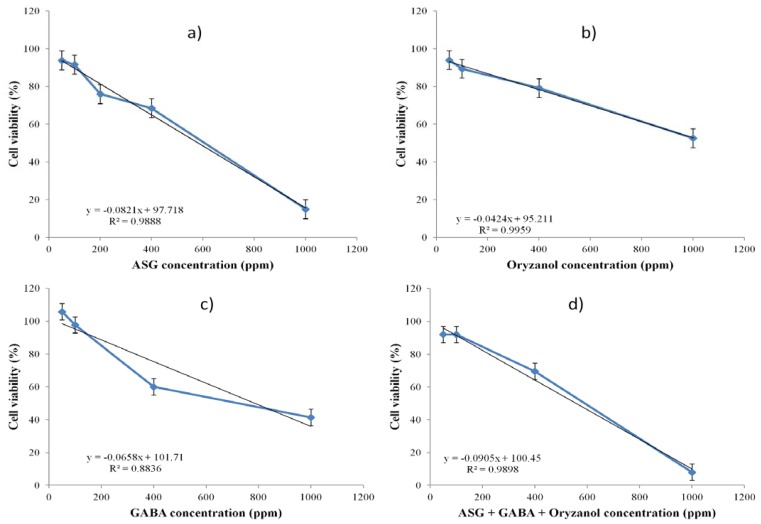
Effects of (**a**) acylated steryl glycoside (ASG), (**b**) oryzanol, (**c**) gamma amino butyric acid (GABA), and (**d**) ASG + GABA + Oryzanol, on HEP-G2 cell viability. HEP-G2 cells treated with ASG, GABA and oryzanol were incubated for 24 h, after which cell viability was determined. Each data point and the error bars on the graph represent mean ± standard deviation (*n* = 4). Linear regression analyses (represented by the black lines and equations on each graph) show a strong correlation between the concentrations of the extracts and cell viability data obtained from the experiment, as suggested by the high *R*^2^ values.

Cell viability was assessed following the method described by Mosmann (1983) [18]. Briefly, HEP-G2 cells were seeded into a 96-well plate at a density of 1 × 10^5^ cells/well and cultured for 24 h at 37 °C with 5% CO_2_. Cells were then treated with 50–1000 ppm of the extracts for 24 h and subsequently stained with MTT and the resultant chromogen formazen solubilized with DMSO. Absorbance was read at 570 nm in a microplate reader and the cell viability expressed as percentage of live cells relative to controls. The MTT assay results are shown on Figure 1. 

### 2.6. Treatment of HEP-G2 Cells

Cells subcultured on a 24-well plate were allowed to attach for 24 h, after which they were subjected to serum starvation in RPMI 1640 medium supplemented with 0.5% FBS and 1% antibiotics for another 12 h. Starved HEP-G2 cells were treated with non-toxic doses (50 ppm and 100 ppm) of ASG (IC50—581 ppm), GABA (IC50—785 ppm) and oryzanol (IC50—1066 ppm) and insulin (100 nM), in a medium that contained 1 μM dexamethasone, 10% FBS and 1% antibiotic. Also, the effects of ASG, GABA and oryzanol in combination (IC50—653 ppm) were studied. Following 24 h of treatment, the medium was removed and RNA extracted after washing cells with PBS.

### 2.7. Gene Expression Study

Primers were designed on NCBI website except for internal control (Kanr), which was supplied by Beckman Coulter (Miami, FL, USA). Primers shown on Table 1 were supplied by First base (Selangor, Malaysia) and reconstituted in 1× TE buffer to final concentrations of 200 nM and 500 nM for forward and reverse primer sets, respectively. RNA was extracted from HEP-G2 cells at the end of cell culture study using the Total RNA Isolation kit (RBC Bioscience Corp., Taipei, Taiwan), according to the manufacturer’s instructions. Reverse transcription and PCR were done according to the GenomeLab™ GeXP Kit (Beckman Coulter, Miami, FL, USA) protocol, in an XP Thermal Cycler (Bioer Technology, Germany). PCR products were finally analyzed on the GeXP genetic analysis system and results normalized on eXpress Profiler software based on the manufacturer’s instructions.

**Table 1:** Gene name and sequences of primers used in multiplex panel.

Gene name	Forward primer sequence	Reverse primer sequence
Insulin Receptor	AGGTGACACTATAGAATAAAGACAGTGAGCTGTTCGAGC	GTACGACTCACTATAGGGAAGTGCCTGAAGAGGTTTTTCTG
3-hydroxy-3-methyl-glutaryl-CoA reductase	AGGTGACACTATAGAATAAATGGCAACAACAGAAGGTTGT	GTACGACTCACTATAGGGAGAAACGGATATAAAGGTTGCGT
Peroxisome proliferator-activated receptor gamma	AGGTGACACTATAGAATACAGAAATGACCATGGTTGACA	GTACGACTCACTATAGGGAGGCTCTTCATGAGGCTTATTG
Peptidylprolyl isomerase A *^,#^	AGGTGACACTATAGAATACACACGGCTCACATTGCAT	GTACGACTCACTATAGGGACACGAACAGCAAAGCGA
Beta actin *	AGGTGACACTATAGAATAGATCATTGCTCCTCCTGAGC	GTACGACTCACTATAGGGAAAAGCCATGCCAATCTCATC
Glyceraldehyde-3-phosphate dehydrogenase *	AGGTGACACTATAGAATAAAGGTGAAGGTCGGAGTCAA	GTACGACTCACTATAGGGAGATCTCGCTCCTGGAAGATG

* Housekeeping genes. ^#^ Used for normalization.

### 2.8. Statistical Analysis

Means of groups were used in the analyses (*n* = 4) and, where error bars are shown, they represent standard deviation. One-way analysis of variance (ANOVA) on SPSS 17.0 software (SPSS Inc., Chicago, IL, USA) was used to assess the level of significance between means at *p* < 0.05. Linear regression analysis was conducted on the MTT results to determine the correlation between the concentrations of the extracts used to treat the cells and cell viability due to such treatments (Figure 1).

## 3. Results and Discussion

### 3.1. Analysis of Extracts

Despite reports of improved metabolic indices by GBR, the contribution of its bioactive compounds towards such effects and mechanisms of action have largely not been reported. However, its functional effects are likely a result of synergy from its bioactives. GABA, oryzanol, and ASG have been reported to possess beneficial health effects, and, in fact, several studies have tried to potentiate the concentration of some of these bioactives in order to maximize their benefits [1,2]. In the current study, our GBR variety was found to be bioactive rich, with a GABA concentration of 0.36 ± 0.04 mg/g GBR and ASG of 0.465 ± 0.055 mg/g GBR. Four isomers of oryzanol (cycloartenyl ferulate, 24-methylene cycloartanyl ferulate, campestryl ferulate and mixtures of β-sitosteryl ferulate and cycloartanyl ferulate) were found to be in the range of 30.38–64.22 mg/g GBR. Our GBR variety may therefore be a rich source of these bioactives and as dietary components or nutraceuticals they may provide enormous benefits in view of their reported potentials. 

### 3.2. HEP-G2 PPARγ Gene Expression

PPARγ is known to regulate a wide variety of metabolic pathways at transcriptional level with effects including those of glucose metabolism, lipoprotein metabolism, cholesterol synthesis and adipocytokines [4]. Breakdown in regulation of *PPARγ* could affect a wide range of pathways, and bioactive compounds can influence such regulation [7,11,19]. Interestingly, however, the antiobesity effect of GBR was reported to involve downregulation of the *PPARγ* [10], in contrast to findings that upregulation of the *PPARγ* improved metabolic outcomes. This suggests that the *PPARγ* has varied effects and regulates multiple pathways, and it appears that a balance is needed in its transcriptional activity to maintain optimal metabolic regulation of all pathways affected by PPARγ. Also, it could be argued that the antiobesity effects of GBR may have been due to multiple factors affecting many more pathways, in addition to downregulation of the *PPARγ*, in view of the many bioactive compounds present in GBR [1,2]. 

**Figure 2:**
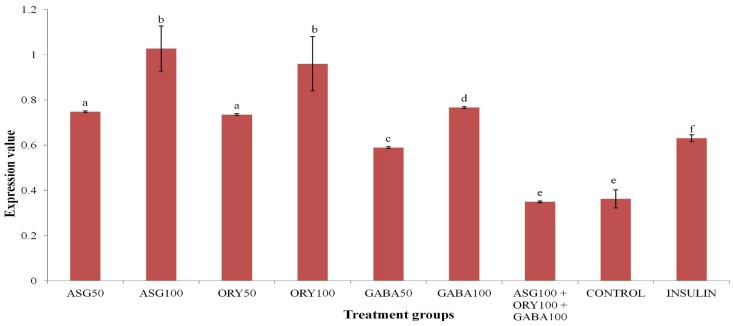
Expression of HEP-G2 peroxisome proliferator-activated receptor gamma (*PPARγ*) gene; treatment of HEP-G2 cells with 50 ppm and 100 ppm of extracts showed upregulation of the gene, above normal (insulin) and control, to varying degrees. All extracts significantly upregulated the expression of the gene in comparison to control (*p* < 0.05) in a dose-dependent manner (*n* = 4), but when combined (100 ppm), they downregulated the gene. Bars with different letters are significantly different (*p* < 0.05). ASG-acylated steryl glycoside, ORY-oryzanol, GABA-gamma aminobutyric acid.

We studied the effects of oryzanol, GABA and ASG on expression of *PPARγ*, *HMGCR* and *IR* genes on a multiplex panel, in order to determine the effects of extracts, if any, on key aspects of cholesterol metabolism (*HMGCR*) and insulin signaling (*IR*) in addition to *PPARγ* modulation. There were no significant effects on *HMGCR* and *IR* mRNA levels with all our treatments. However, all three bioactive extracts showed a dose dependent increase in mRNA levels of *PPARγ*, significantly higher than control and insulin treatments (Figure 2). At 50 ppm, oryzanol and ASG increased *PPARγ* mRNA levels significantly higher than insulin, while GABA did not. At 100 ppm, however, all three extracts increased expression of *PPARγ* more than insulin did. Interestingly, the treatment of HEP-G2 cells with all three extracts (100 ppm) resulted in downregulation of the gene, in agreement with the findings by Ho *et al.* [10].

Insulin has been reported to significantly influence the expression of *PPARγ* [20]. In the presence of insulin, *PPARγ* expression increases beyond basal levels, and our results also show this effect. In the presence of insulin, expression of *PPARγ* was almost twice as much as in control group likely reflecting normal physiological conditions in biological systems. Furthermore, it is expected that insulin resistance triggers release of more insulin as a way of overcoming insulin resistance through higher concentrations. This partly explains how the effect of insulin on *PPARγ* may be involved in improving deranged metabolic indices in insulin-resistant conditions. However, in view of the effect of GABA, ASG, and oryzanol on expression of *PPARγ*, it is likely that they may be more effective than insulin in improving metabolic indices in biological systems even in insulin-resistant conditions. Furthermore, our results show that GABA, oryzanol, and ASG likely contribute towards downregulation of the *PPARγ* when GBR is consumed. Through upregulation of the *PPARγ* and subsequent effects on downstream regulators of insulin sensitivity, glucose metabolism, and other indices, GABA, ASG, and oryzanol could improve metabolic states in which perturbations of *PPARγ* are implicated. On the other hand, GBR as a whole may be more useful in conditions like obesity, where downregulation of *PPARγ* is beneficial.

The current findings may have a wide range of implications. More studies of the effects of the individual bioactives of GBR like GABA, oryzanol, and ASG, or even GBR, as a whole, may provide better understanding of their potential therapeutic value in conditions where *PPARγ* dysregulation is the underlying cause. These bioactive compounds and/or GBR may produce varied metabolic responses, such as remodeling of cellular biology to reduce insulin insensitivity, inflammation, and other metabolic disturbances originating from dysregulated cellular functioning. What is promising, however, is that GBR and its bioactive compounds may offer metabolic improvements in a wide range of disease conditions, including type 2 diabetes, atherosclerosis, different cancers, obesity, and other conditions [5,7,10,19]. Despite their different pathophysiologies, the abilities of GBR and its bioactive compounds to have varying and even opposite effects on *PPARγ*, which is a common denominator for many chronic conditions, present an interesting lead for future studies. These studies may provide better understanding on how these dietary components elicit their effects and in which conditions they may be of therapeutical value. 

## 4. Conclusions

GBR is known to improve several metabolic indices, though the mechanisms of action involved are not fully understood. *PPARγ* upregulation has been reported to improve metabolic indices in type 2 diabetes and other chronic conditions, while its downregulation has been shown to confer antiobesity effects. Our findings suggest that GABA, oryzanol, and ASG present in GBR may individually upregulate the *PPARγ*, with the potential to improve several metabolic conditions. On the other hand, when combined, the bioactive compounds downregulated the *PPARγ*, an effect similar to what whole GBR is known to elicit as part of its antiobesity effects. GBR and its bioactive compounds, therefore, have varied and interesting effects on the *PPARγ*, which need to be studied further for their potential therapeutic value in a wide range of metabolic conditions. 
